# Dietary Fat and Protein Intake in Relation to Plasma Sphingolipids as Determined by a Large-Scale Lipidomic Analysis

**DOI:** 10.3390/metabo11020093

**Published:** 2021-02-08

**Authors:** Jowy Yi Hoong Seah, Wee Siong Chew, Federico Torta, Chin Meng Khoo, Markus R. Wenk, Deron R. Herr, E. Shyong Tai, Rob M. van Dam

**Affiliations:** 1 Saw Swee Hock School of Public Health, National University of Singapore (NUS), Singapore 117549, Singapore; tai_e_shyong@nuhs.edu.sg; 2 NUS Graduate School for Integrative Sciences and Engineering, NUS, Singapore 119077, Singapore; 3 Department of Pharmacology, Yong Loo Lin School of Medicine, NUS, Singapore 117600, Singapore; wuweisiong@yahoo.com (W.S.C.); phcdrh@nus.edu.sg (D.R.H.); 4 Department of Biochemistry, Yong Loo Lin School of Medicine, NUS, Singapore 117596, Singapore; bchfdtt@nus.edu.sg (F.T.); bchmrw@nus.edu.sg (M.R.W.); 5 Singapore Lipidomics Incubator, Life Sciences Institute, NUS, Singapore 117456, Singapore; 6 Department of Medicine, Yong Loo Lin School of Medicine, NUS and National University Health System, Singapore 119228, Singapore; mdckcm@nus.edu.sg; 7 Department of Biology, San Diego State University, San Diego, CA 92182, USA; 8 Duke-NUS Graduate Medical School, Singapore 169857, Singapore; 9 Department of Nutrition, Harvard T.H. Chan School of Public Health, Boston, MA 02115, USA

**Keywords:** ceramides, hexosylceramides, lipidomics, monounsaturated fat, protein, polyunsaturated fat, saturated fat, sphingomyelin

## Abstract

Sphingolipid concentrations have been associated with risk of type 2 diabetes and cardiovascular diseases. Because sphingolipids can be synthesized de novo from saturated fatty acids (SFA), dietary fatty acids may affect plasma sphingolipid concentrations. We aimed to evaluate dietary fat and protein intakes in relation to circulating sphingolipid levels. We used cross-sectional data from 2860 ethnic Chinese Singaporeans collected from 2004–2007. Nutrient intakes were estimated on the basis of a validated 159-item food frequency questionnaire. We quantified 79 molecularly distinct sphingolipids in a large-scale lipidomic evaluation from plasma samples. Higher saturated fat intake was associated with higher concentrations of 16:1;O2 sphingolipids including ceramides, monohexosylcermides, dihexosylceramides, sphingomyelins, and sphingosine 1-phosphates. Higher polyunsaturated fat intake was associated with lower plasma long-chain ceramides and long-chain monohexosylcermide concentrations. Protein intake was inversely associated with concentrations of most subclasses of sphingolipids, with the exception of sphingolipids containing a 16:1;O2 sphingoid base. Lower intake of saturated fat and higher intake of polyunsaturated fat and protein may decrease plasma concentrations of several sphingolipid classes. These findings may represent a novel biological mechanism for the impact of nutrient intakes on cardio-metabolic health.

## 1. Introduction

Sphingolipids are a molecularly diverse type of lipid with a wide variety of biological functions in eukaryotic cells. They differ by head group structure, chain length, and unsaturation degree in the sphingoid backbone and fatty acyl chain. Certain classes of sphingolipids have emerged as novel biomarkers of metabolic disorders and chronic diseases. In prospective cohort studies, sphingomyelin (SM) species were directly associated with risk of type 2 diabetes (T2D) [1] and cardiovascular diseases (CVDs) [2,3,4]. Higher concentrations of hexosylceramides and ceramides were also associated with higher CVD risk [2,3,4,5,6,7], although findings varied across studies. Several mechanisms have been proposed for a role of plasma sphingolipids, particularly ceramides, in the pathogenesis of chronic diseases. Ceramides may increase chronic disease risk by promoting inflammation [8,9,10]. In cell studies, endogenous ceramides promoted low-density lipoprotein (LDL) uptake [11] and transcytosis of oxidized LDL [12], and both events may play a role in atherosclerosis. In addition, ceramides and complex sphingolipids derived from ceramides, such as glucosylceramides (GluCers), may act as mediators of cell death induction [13] and promote T2D through inducing pancreatic beta-cell toxicity and apoptosis, resulting in dysfunctional insulin secretion [14,15].

Because sphingolipids can be synthesized de novo from saturated fatty acids (SFAs) that are incorporated as a long chain base [16], it is biologically plausible that higher intakes of SFA will result in higher circulating sphingolipid concentrations. In two feeding trials, overeating diets high in SFA, but not polyunsaturated fatty acid (PUFA), increased plasma ceramide concentrations [17,18]. While there were other trials that evaluated the effects of specific foods or dietary patterns on circulating ceramides or SM, results have been inconsistent [6,19,20,21,22]. For instance, in a randomized Finnish trial, fatty fish intake decreased ceramide concentrations, but in a double-blinded randomized Norwegian trial [20], fish oil supplementation did not affect circulating ceramides [22]. It is difficult to ascertain specific aspects of the foods or diets that may have led to the discrepancies. Furthermore, only a limited number of ceramide or sphingomyelin species were measured in these trials. No studies on dietary determinants of other classes of sphingolipids such as hexosylceramides are available and data from free-living populations are lacking. We addressed this gap by examining associations between fatty acid and protein intake and 79 circulating sphingolipid levels measured through a large-scale lipidomic analysis in a population-based cohort in Singapore.

## 2. Results

Baseline characteristics of the participants are shown in Table 1. The participants had a mean age of 48.6 (SD 12.0) years and a mean body mass index (BMI) of 22.9 (SD 3.8) kg/m^2^. About half (47.0%) of the participants were male. Average concentrations of the quantified sphingolipids are listed in Supplemental Table S1.

**Figure 1:**
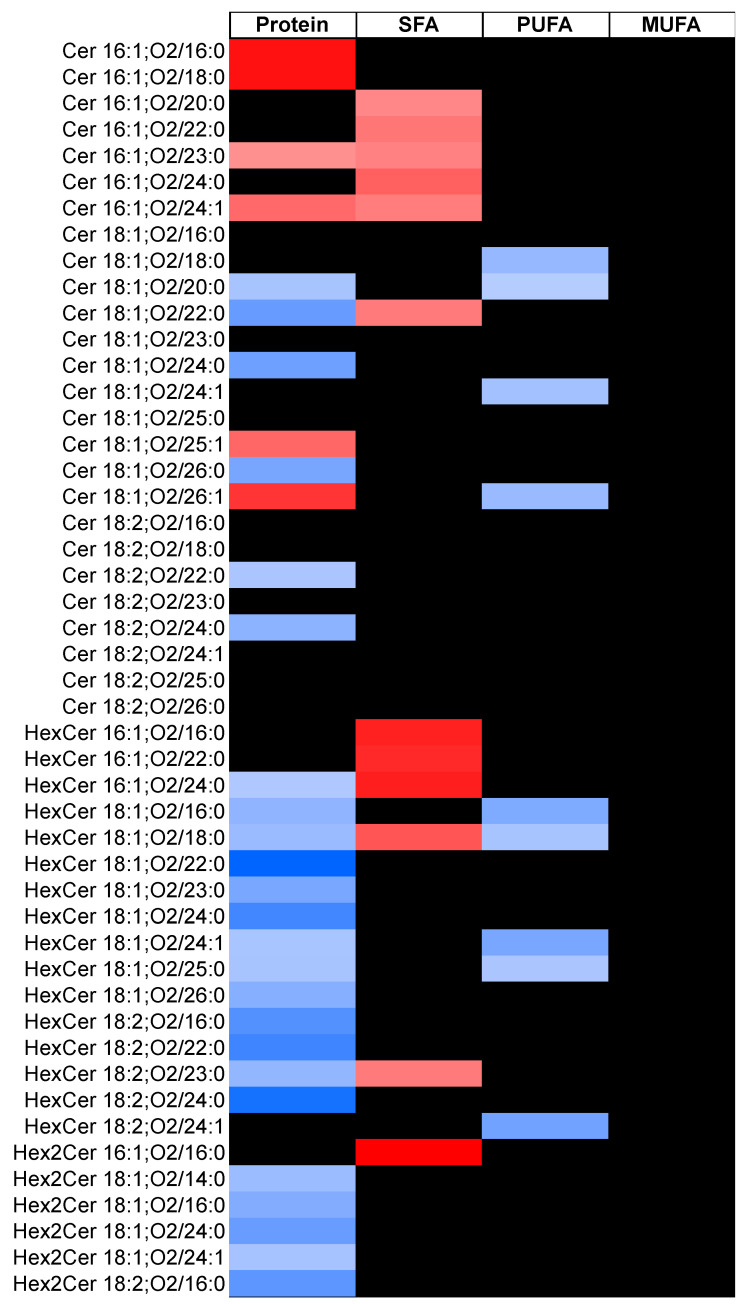

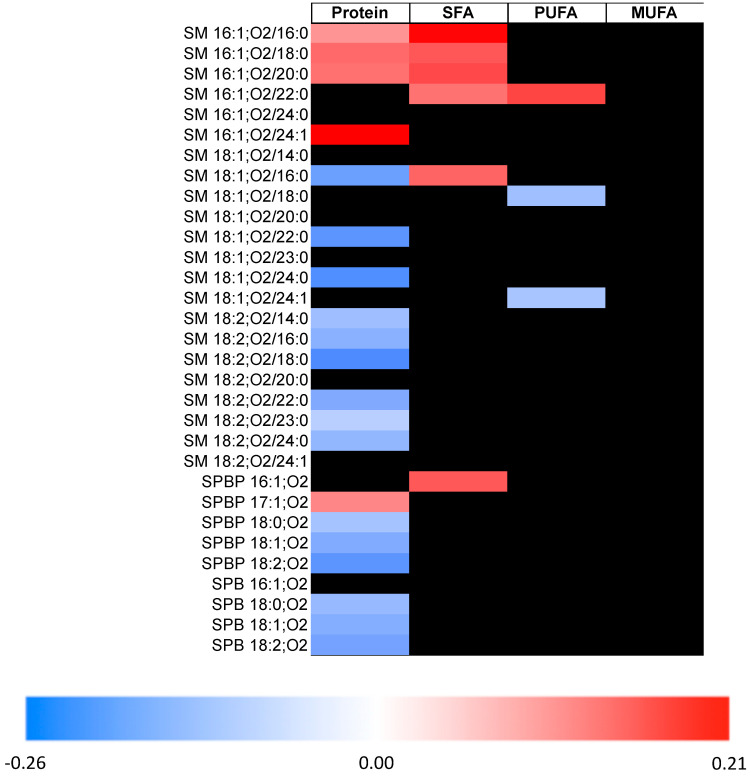
Associations between intakes of protein and different types of fat, and individual sphingolipid concentrations of the SP2 participants (*n* = 2860). Associations that are not statistically significant (adjusted *p* > 0.050) after Benjamini‑Hochberg correction are indicated with a black box. We quantified 26 ceramides (Cers), 16 monohexosylceramides (HexCers), 6 dihexosylceramides (Hex2Cers), 22 sphingomyelins (SMs), 5 sphingoid base phosphates (SPBPs), and 4 sphingoid bases (SPBs) for a total of 79 sphingolipid species. The colors are beta coefficients from linear regression analysis representing the change per SD in circulating sphingolipids (log-transformed values) for every 5% of total energy intake increment in nutrient intake. We adjusted for age, sex, energy intake, BMI, alcohol intake, physical activity, cigarette smoking, high-density lipoprotein cholesterol, low-density lipoprotein cholesterol, and triglycerides. We adjusted for age, sex, energy intake, BMI, alcohol intake, physical activity, cigarette smoking, high-density lipoprotein cholesterol, low-density lipoprotein cholesterol, and triglycerides. Additionally, for protein and total fat outcomes, we mutually adjusted them for each other, and for saturated fat, polyunsaturated fat, and monounsaturated fat outcomes, these were mutually adjusted for each other in addition to protein intake. Abbreviations: MUFA, monounsaturated fatty acid, PUFA, polyunsaturated fatty acid, SFA, saturated fatty acid.

Table 2 shows associations between intakes of protein and different types of fat, and classes and subclasses of circulating sphingolipids. Higher SFA intake was associated with higher concentrations of several classes of sphingolipids, with a 16:1;O2 backbone including ceramides, monohexosylceramide (HexCer), dihexosylceramide (Hex2Cer), SM, and sphingoid base phosphate (SPBP). Higher SFA consumption was also associated with higher concentrations of long-chain SM. We obtained similar results when we evaluated associations between SFA intake and individual sphingolipids (Figure 1 and Supplemental Tables S2–S5); higher SFA intake was associated with higher levels of 18 species of sphingolipids, and 13 of these contained a 16:1;O2 sphingoid backbone.

Higher PUFA intake was associated with lower long-chain ceramides and long-chain HexCer concentrations (Table 2). Among individual sphingolipids, higher PUFA intake was associated with lower concentrations of four species of ceramides, five species of HexCer, and two species of SM (Figure 1). PUFA intake was also directly associated with SM 16:1;O2/22:0. Monounsaturated fatty acid (MUFA) intake was not associated with plasma sphingolipid concentrations.

Higher intakes of protein were associated with lower concentrations of all classes of sphingolipids including ceramides, HexCer, Hex2Cer, SM, sphingoid base (SPB), and SPBP (Table 2). Among individual sphingolipids, protein intake was inversely associated with 6 species of ceramide, 13 species of HexCer, 5 species of Hex2Cer, 9 species of SM, 4 species of SPBP, and 3 species of SPB (Figure 1). Notably, of these 40 species, only 1 species (HexCer 16:1;O2/24:0) contained a 16:1;O2 sphingoid backbone. In contrast, protein intake was directly associated with six species of ceramides, four species of SM, and one SPBP species. Out of 11 species, 8 contained a 16:1;O2 sphingoid backbone.

## 3. Discussion

In this large cross-sectional study of 2860 ethnic Chinese participants, we examined associations between nutrient intake and sphingolipid concentrations. Higher SFA intake was associated with higher concentrations of 16:1;O2 sphingolipids, including ceramides, HexCer, Hex2Cer, SM, and SPBP. Higher PUFA intake was associated with lower plasma long-chain ceramides and long-chain HexCer concentrations. Higher protein intake was associated with lower concentrations of most subclasses of sphingolipids, except for 16:1;O2 sphingolipids.

The first step of sphingolipid de novo synthesis involves the formation of 3-keto-dihydrosphingosine from serine and fatty acyl-CoA (mostly palmitoyl-CoA). This rate-limiting reaction is catalyzed by the heterotrimeric enzyme serine palmitoyltransferase (SPT) [16]. The 3-keto-dihydrosphingosine undergoes further reactions to form ceramide. Ceramides are the precursors of various diverging sphingolipid metabolic pathways. Ceramides may undergo glycosylation to more complex sphingolipids by the addition of a monosaccharide resulting in HexCer [16]. This can include sugar moieties such as glucose or galactose, which results in GluCer or galactosylceramide (GalCer), respectively. The addition of a second monosaccharide to HexCer or the addition of a disaccharide to ceramides forms Hex2Cer [16]. Ceramides can also react with phosphatidylcholine to generate SM [16]. Finally, ceramides may be converted to sphingosine and S1P (an SPBP) [16], a pathway that is marked for catabolism.

Although palmitoyl-CoA is the typical precursor for endogenous synthesis, resulting ultimately in 18:1;O2 sphingolipids, there are heterodimers of serine palmitoyltransferase that show preference for other fatty acyl-CoA precursors, such as myristate-CoA (C14-CoA) that will lead to 16:1;O2 sphingolipids [23]. Thus, the direct association between SFA intake and 16:1;O2 sphingolipid levels in our study may have resulted from endogenous conversion of C14-CoA to 16:1;O2 ceramides, which are then further transformed to other 16:1;O2 sphingolipids. This hypothesis is supported by three premises: first, the sphingoid backbone in de novo synthesis is conserved [16]; second, we observed generally consistent direct associations between SFA intake and 16:1;O2 sphingolipids; third, in animal studies, SPT levels were increased following diets high in SFA [24]. In an animal study, higher levels of 16:1;O2 sphingolipids resulting from dietary myristate promoted cardiomyocyte apoptosis, suggesting that high SFA intake in the form of myristate was not beneficial to cardiovascular health [25].

Because sphingolipids can also be found in foods [26,27], we cannot exclude the possibility that our results may have reflected dietary contribution of pre-formed sphingolipids rather than de novo synthesis. However, this contribution is unlikely to be substantial, particularly for complex sphingolipids such as SM and ceramides that must be broken down into simpler sphingolipids and subsequently absorbed as sphingosines [27,28]. Currently, data from human studies on dietary absorption of other pre-formed sphingolipids are lacking.

In comparison with SFA intake, it is currently less clear what mechanism may underpin the inverse associations between PUFA intake and long-chain ceramides and long-chain HexCer. Although PUFA-rich diets increased SPT in rats, these diets also reduced circulating SM-ase activity [24] and possibly ceramidase activity [24,29]. Increased SPT activity may contribute to higher ceramide concentrations through de novo synthesis, whereas decreased SM-ase and increased ceramidase activity may lower ceramide levels by downregulation of hydrolysis of SM to ceramides and upregulation of catalytic cleavage of ceramides, respectively [24,29]. Thus, the overall changes in ceramide levels resulting from PUFA intake may be due to the competing effects of changes in SPT, SMase, and ceramidase. Our findings for protein intake and sphingolipid concentrations are novel and require further mechanistic research.

In a longitudinal crossover trial, muscle ceramide concentrations in patients with T2D were not significantly different following a fat-rich diet and a carbohydrate-rich diet [30]. Participants lost weight after both diets, which complicates the interpretation of the results. However, the lack of effects on ceramide concentrations in this trial are consistent with our finding that the type of fat and amount of protein in the diet may be more relevant than total fat and carbohydrate intakes. The effects of overfeeding SFA and PUFA on ceramide concentrations was examined in two randomized trials of over-feeding. In a Swedish trial, SFA intake in the form of palm oil (mainly palmitate, 16:0) significantly increased C16:0 GluCer, C16:0 Hex2Cer, C18:0 ceramides, C20:0 ceramides, C24:0 ceramides, and C24:1 ceramides in comparison with PUFA (administered as sunflower oil, mainly linoleate, 18:2n−6) [18]. In a Finnish trial, dietary SFA (as coconut oil, butter, and cheese) significantly increased circulating Cer 18:0;O2/24:0, Cer 18:1;O2/24:0, Cer 18:1;O2/24:1, and Cer 18:2;O2/23:0 [17], whereas dietary PUFA (as olive oil, pesto, and nuts) did not significantly change ceramide concentrations [17]. Results from the Swedish and Finnish trials are therefore consistent with our finding that higher SFA intake, but not PUFA intake, is associated with higher ceramide and hexosylceramide concentrations. However, the exact subclasses and species affected by high SFA intake in these trials were different from our study and a number of reasons may explain these discrepancies. First, differences in the fatty acid composition of the dietary SFA may have driven the synthesis of ceramides with different sphingoid backbones. Feeding of SFA high in myristic acid (14:0) should result in 16:1;O2 sphingolipids whereas feeding palmitic acid (16:0) should result in 18:1;O2 sphingolipids. Second, there may have been differences in laboratory methods used to extract and quantify the ceramides and the specific species quantified were different. For instance, we measured 16:1;O2 and 18:x/O2 sphingolipids on a large scale but the Finnish trial only focused on a small number of 18:x/O2 sphingolipids [17]. Finally, the trials examined the effects of overfeeding, and the results may thus have been affected by excess energy intake.

Several studies have documented the association between circulating sphingolipids and risk of chronic diseases. In our cohort, we observed direct associations between species of SM and risk of T2D [1], and between HexCer, Hex2Cer, and SM and risk of CVD [2]. In other prospective studies, higher concentrations of certain ceramides, HexCer, and SM were associated with higher risk of CVD [5,6], heart failure [3], or CVD mortality [4], although the results differed on the basis of the subclasses of sphingolipids analyzed. Our results therefore suggest that substituting SFA intake with other macronutrients, particularly PUFA, may decrease concentrations of several sphingolipid classes involved in the pathogenesis of chronic diseases.

We used validated methods to measure our exposures and outcomes: a detailed interviewer-administered food frequency questionnaire (FFQ) [31] and published techniques for sphingolipid extraction and quantification [32,33]. We also acknowledge several potential limitations. First, our assessment of diet in terms of self-reports may have resulted in measurement errors, although this would have attenuated the observed associations. Second, although we adjusted for a range of potential confounders, we cannot fully exclude the possibility of residual confounding by unmeasured or imperfectly measured factors. Third, inferring causality is difficult in cross-sectional studies, although it is unlikely that the participants were aware of their plasma sphingolipid profile and that this would have influenced their dietary choices. Fourth, we only measured sphingolipid concentrations in plasma, and this may not be reflective of sphingolipid levels in different tissues. Finally, caution is needed in generalizing our findings to populations of a different ethnicity.

## 4. Materials and Methods

### 4.1. Study Population

The participants used in our cross-sectional analysis were selected from the Singapore Prospective Study Program, a population-based study conducted in adult Singaporeans between 2004 and 2007. The methods of this study have previously been described [2,34]. In brief, all participants were from 4 previous representative population-based studies. Upon agreeing to be interviewed in their homes, participants had their personal information in terms of socio-demographic and lifestyle factors and medical history collected using standardized questionnaires. Following the interviews, participants were invited to attend a health examination for physical measurements and collection of blood samples after fasting for at least 8 h. For the current project, we focused on the 2998 ethnic Chinese participants with blood samples because sphingolipid levels are known to differ by ethnic groups [35,36]. After excluding participants with history of heart attack (*n* = 24), stroke (*n* = 26) or cancer (*n* = 35), and statin use (*n* = 61), we resulted in 2860 participants for the current analysis. Reasons for exclusion were not mutually exclusive. Ethics approval was obtained from the institutional review boards of the National University of Singapore and Singapore General Hospital (CIRB ref #2001/001/C). We obtained informed consent from all participants before the study was conducted.

### 4.2. Lipid Extraction and Quantification

Butanol/methanol (1:1) solvent containing internal standards was used to extract lipids from plasma samples [32]. For sphingosine 1-phosphates (S1P), we additionally performed a trimethylsilyl-diazomethane derivatization step to improve sensitivity [33]. Lipids were separated on a ultra high performance liquid chromatography Agilent 1290(Agilent Technologies, Santa Clara, USA), using a Waters BEH HILIC (Milford, MA, USA) 100 mm column (for S1P) or a reversed phase Agilent ZORBAX RRHD Eclipse Plus(Agilent Technologies, Santa Clara, USA) C18 50 mm column (for all other sphingolipids), and were analyzed by positive mode electrospray ionization mass spectrometry using an Agilent 6495 QQQ mass spectrometer (Agilent Technologies, Santa Clara, USA). Quantification was performed using a dynamic multiple reaction monitoring method with measurement of peak area of quantifier transitions by peak integration. Lipid peaks were identified on the basis of their specific precursor > product ion transitions and retention time [32]. Thereafter, normalization with internal standards was carried out [32]. Pooled quality control (QC) samples were included every 10 study samples to ensure the quality and precision of the results. We excluded lipids that showed coefficient of variation (CV) higher than 30% in QC samples or a low signal-to-noise ratio (S/N < 5). An upper limit of 30% for the CV is generally accepted for the analysis of metabolomics biomarkers [37,38,39]. A total of 331 peaks were evaluated for each plasma sample and we subsequently included 79 peaks that met our inclusion criteria for reliability in subsequent analyses. These peaks included 26 ceramides, 16 cerebrosides (monohexosylceramides, HexCers), 6 globosides (dihexosylceramides, Hex2Cers), 22 sphingomyelins (SMs), 5 SPBPs, and 4 sphingoid bases (SPBs). These lipids are described using updated nomenclature guidelines [40]. We used principal component analysis plots of the relative abundance of the lipids in the samples and QCs to detect any potential drifts and batch effects. As is often the case for lipidomic measurements, we used one internal standard for each lipid class, as our goal was to perform relative quantification as opposed to absolute quantification of the endogenous species [1].

### 4.3. Assessment of Diet and Covariates

A trained interviewer obtained dietary information over the past month using a 159-item semi-quantitative food frequency questionnaire (FFQ) at the participants’ homes. The respondent was asked to report intake for each of the food items as frequency per day, per week, per month, or rarely/never. Nutrient and energy intakes were then calculated by the Singapore Health Promotion Board using an in-house database with information on energy and nutrient values of local foods. On the basis of the frequency of consumption, weight of the food item consumed, and the nutrient composition, we calculated the amount of energy and each of the nutrients contributed by each food item. We validated the FFQ against three 24-h recalls and obtained reasonably good correlation coefficients for energy and nutrient intakes ranging from 0.46 to 0.58 [31].

Physical activity in the leisure time, occupational, household, and transport domain were assessed using a validated questionnaire [41] and metabolic equivalent task (MET)-hours per week were calculated [41]. Other covariables including age, sex, cigarette smoking, and alcohol intake were also assessed with the baseline questionnaire, while height and weight were collected during the physical examination.

We calculated body mass index (BMI) by taking the weight (kg) of participants divided by the square of their height (m^2^). We used standard enzymatic assays to determine LDL cholesterol, high-density lipoprotein (HDL) cholesterol, and triglycerides from fasting blood samples [42].

### 4.4. Statistical Analyses

Intakes of SFA, PUFA, MUFA, and protein were expressed as percentage of total energy intake. Aggregated lipid concentrations were calculated by taking the sum of individual lipids according to lipid class, *N*-acyl chain length, and sphingoid backbone. All lipid variables were log-transformed before analyses. We truncated any values not within mean ± 4 SD of the log-transformed variables to improve normality.

Multivariable linear regression was used to model associations between nutrient intakes (SFA, PUFA, MUFA, and protein, expressed as 5% of total energy intake increment) and plasma sphingolipids (expressed as per SD increment of the log-transformed values). We considered the following potential confounding variables: age at recruitment (years), sex, cigarette smoking (never smoker, ex-smoker, current smoker), alcohol intake (never or hardly ever, mild to moderate (>0 to ≤2 drinks/d for males and >0 to ≤1.5 drinks/d for females), moderate to heavy (>2 to <3.5 drinks/d for males and >2 to <2.5 drinks/d for females), heavy (≥3.5 drinks/d for males and ≥2.5 drinks/d for females)), energy intake (kcal/d), nutrient intakes (SFA, PUFA, MUFA, and protein, as percentage of energy intake), HDL cholesterol (mmol/L), LDL cholesterol (mmol/L), and triglycerides (mmol/L). These covariates were selected a priori on the basis of the literature on potential determinants of plasma sphingolipids, as well as on our previous publication involving participants of the same cohort where we examined relationships between cardiovascular risk factors and circulating sphingolipids [1]. The multivariable models included total energy intake and other macronutrients but excluded carbohydrate intakes. Hence, total energy intake was “kept constant”, and the presented regression coefficients for SFA, PUFA, MUFA, and protein represented isocaloric substitutions with carbohydrate. For instance, β coefficients for polyunsaturated fat when total energy intake, saturated fat, monounsaturated fat, and protein intakes were included in the multivariable model represented the change in sphingolipid concentrations for a replacement of 5% of energy from carbohydrate with 5% of energy from polyunsaturated fat. Carbohydrate was therefore the “reference” nutrient.

We used Stata Software version 14 (StataCorp LP, College Station, TX, USA) for all statistical analyses. We corrected for multiple testing using the Benjamini–Hochberg method [43], and adjusted *p*-values < 0.050 were considered statistically significant.

## 5. Conclusions

In our population-based study, higher SFA intake was associated with higher plasma concentrations of 16:1:O2 ceramides, HexCer, Hex2Cer, SM, and SPBP. In contrast, PUFA intake was associated with lower long-chain ceramides and long-chain HexCer concentrations, and protein intake was associated with lower concentrations of most subclasses of sphingolipids. Higher intakes of SFA, and lower intakes of PUFA and protein may increase classes of circulating sphingolipids associated with the development of T2D and CVD. Therefore, effects on sphingolipid profiles may represent a novel biological pathway for the impact of nutrient intakes on risk of cardio-metabolic diseases.

## Figures and Tables

**Table 1:** Baseline characteristics of study population (*n* = 2860) ^1^.

Age at interview (y)	48.6 ± 12.0
Male (*n* (%))	1345 (47.0)
Cigarette smoking (*n* (%))	
Never-smoker	2295 (80.2)
Ex-smoker	247 (8.6)
Current smoker	318 (11.1)
Alcohol intake (*n* (%))	
Never or hardly ever	2210 (77.3)
Mild to moderate	220 (7.7)
Moderate to heavy	159 (5.6)
Heavy	271 (9.5)
Physical activity (MET-h/week) ^2^	108 ± 82
Energy intake (kcal/d)	2168 ± 920
Nutrient intake (% energy)	
Protein	15.9 ± 2.2
Total fat	30.5 ± 5.6
Saturated fat	11.4 ± 2.8
Polyunsaturated fat	6.4 ± 2.7
Monounsaturated fat	10.8 ± 2.7
Body mass index (kg/m^2^)	22.9 ± 3.8
HDL cholesterol (mmol/L) ^3^	1.48 ± 0.37
LDL cholesterol (mmol/L) ^3^	3.21 ± 0.81
Triglycerides (mmol/L) ^3^	1.31 ± 0.86

^1^ Mean ± SD (all such values). ^2^ Refers to metabolic equivalent of task-hours per week. ^3^ Log transformed, truncated, and back-transformed.

**Table 2:** Associations between intake of protein and different types of fat, and subclasses of circulating sphingolipids of the Singapore Prospective Study Program participants (*n* = 2860) ^1^.

	Protein			Saturated Fat			Polyunsaturated Fat			Monounsaturated Fat		
	β^2^	95% CI		β	95% CI		β	95% CI		β	95% CI	
**Ceramides**	**−0.11**	**−0.18**	**−0.04**	0.07	0.01	0.14	−0.01	−0.07	0.04	−0.02	−0.09	0.05
Short-chain (C14)	NA											
Long-chain (C16–C18)	0.00	−0.08	0.08	0.02	−0.06	0.10	**−0.11**	**−0.18**	**−0.04**	0.00	−0.09	0.08
Very long chain (C20–C26)	**−0.11**	**−0.18**	**−0.05**	0.08	0.01	0.14	−0.01	−0.07	0.05	−0.02	−0.09	0.05
16:1;O2	0.06	−0.01	0.13	**0.13**	**0.06**	**0.20**	0.05	−0.01	0.12	−0.10	−0.17	−0.03
18:1;O2	**−0.12**	**−0.19**	**−0.06**	0.07	0.00	0.14	−0.03	−0.08	0.03	0.00	−0.07	0.07
18:2;O2	**−0.10**	**−0.18**	**−0.03**	0.04	−0.04	0.11	0.02	−0.05	0.08	−0.06	−0.13	0.02
**HexCer**	**−0.20**	**−0.28**	**−0.12**	0.07	−0.01	0.15	−0.09	−0.16	−0.02	0.03	−0.06	0.11
Short-chain (C14)	NA											
Long-chain (C16–C18)	**−0.14**	**−0.22**	**−0.05**	0.05	−0.03	0.14	**−0.14**	**−0.22**	**−0.07**	0.03	−0.05	0.12
Very long chain (C20–C26)	**−0.21**	**−0.29**	**−0.13**	0.07	−0.01	0.15	−0.08	−0.15	−0.02	0.02	−0.06	0.11
16:1;O2	−0.06	−0.15	0.02	**0.19**	**0.11**	**0.27**	0.02	−0.05	0.09	−0.06	−0.14	0.03
18:1;O2	**−0.20**	**−0.29**	**−0.12**	0.06	−0.02	0.14	−0.10	−0.16	−0.03	0.03	−0.05	0.11
18:2;O2	**−0.20**	**−0.29**	**−0.12**	0.05	−0.03	0.13	−0.08	−0.15	−0.01	0.00	−0.08	0.08
**Hex2Cer**	**−0.15**	**−0.24**	**−0.07**	0.06	−0.02	0.15	0.01	−0.06	0.08	0.01	−0.07	0.10
Short-chain (C14)	**−0.11**	**−0.20**	**−0.03**	0.06	−0.02	0.14	0.05	−0.02	0.12	−0.04	−0.13	0.04
Long-chain (C16–C18)	**−0.15**	**−0.23**	**−0.06**	0.05	−0.03	0.13	0.01	−0.06	0.08	0.01	−0.07	0.10
Very long chain (C20–C26)	**−0.15**	**−0.23**	**−0.06**	0.09	0.01	0.18	−0.01	−0.08	0.06	0.03	−0.06	0.11
16:1;O2	0.00	−0.09	0.08	**0.21**	**0.12**	**0.30**	0.06	−0.01	0.14	−0.09	−0.18	−0.01
18:1;O2	**−0.15**	**−0.24**	**−0.07**	0.05	−0.03	0.14	0.00	−0.07	0.07	0.02	−0.06	0.10
18:2;O2	**−0.18**	**−0.26**	**−0.10**	0.09	0.00	0.17	0.00	−0.07	0.07	0.00	−0.08	0.08
**SM**	**−0.12**	**−0.19**	**−0.05**	**0.09**	**0.02**	**0.16**	−0.03	−0.09	0.02	0.00	−0.07	0.07
Short-chain (C14)	**−0.08**	**−0.15**	**−0.01**	0.05	−0.02	0.12	0.01	−0.05	0.07	−0.07	−0.14	0.00
Long-chain (C16–C18)	**−0.16**	**−0.23**	**−0.08**	**0.14**	**0.07**	**0.22**	−0.08	−0.14	−0.02	−0.01	−0.08	0.06
Very long chain (C20–C26)	**−0.08**	**−0.15**	**−0.01**	0.03	−0.03	0.10	0.01	−0.05	0.06	0.02	−0.05	0.08
16:1;O2	**0.13**	**0.05**	**0.21**	**0.16**	**0.08**	**0.24**	0.08	0.01	0.14	−0.10	−0.18	−0.02
18:1;O2	**−0.15**	**−0.22**	**−0.08**	0.08	0.01	0.15	−0.06	−0.12	0.00	0.03	−0.04	0.10
18:2;O2	**−0.12**	**−0.19**	**−0.05**	0.03	−0.04	0.09	−0.01	−0.07	0.05	−0.01	−0.08	0.06
**SPB**	**−0.14**	**−0.23**	**−0.05**	0.04	−0.05	0.13	0.00	−0.07	0.08	0.04	−0.05	0.13
16:1;O2	−0.01	−0.10	0.07	0.07	−0.02	0.16	0.04	−0.04	0.11	−0.01	−0.10	0.08
18:1;O2	**−0.14**	**−0.23**	**−0.05**	0.03	−0.06	0.12	−0.01	−0.09	0.07	0.06	−0.04	0.15
18:2;O2	**−0.16**	**−0.25**	**−0.07**	0.03	−0.06	0.12	0.01	−0.07	0.09	0.04	−0.05	0.14
**SPBP**	**−0.15**	**−0.24**	**−0.06**	0.00	−0.09	0.09	0.03	−0.05	0.10	0.09	0.00	0.18
16:1;O2	0.00	−0.09	0.08	**0.14**	**0.05**	**0.23**	0.08	0.00	0.15	−0.02	−0.11	0.07
18:1;O2	**−0.15**	**−0.24**	**−0.06**	0.00	−0.09	0.09	0.02	−0.06	0.10	0.09	0.00	0.18
18:2;O2	**−0.18**	**−0.27**	**−0.09**	−0.03	−0.12	0.06	−0.01	−0.08	0.07	0.11	0.02	0.20

^1^ Values are beta coefficients from linear regression analysis representing the change per SD in circulating sphingolipids (log-transformed values) for every 5% of total energy intake increment in nutrient intake. ^2^ We adjusted for age, sex, energy intake, body mass index (BMI), alcohol intake, physical activity, cigarette smoking, high-density lipoprotein cholesterol, low-density lipoprotein cholesterol, and triglycerides. Additionally, for protein intake, we adjusted for total fat intake, and for saturated fat, polyunsaturated fat, and monounsaturated fat, we mutually adjusted for each other in addition to protein intake. Associations that are statistically significant (adjusted *p* < 0.050, using the Benjamini‑Hochberg procedure) are in bold. Abbreviations: HexCer, monohexosylceramide, Hex2Cer, dihexosylceramide, SM, sphingomyelin, SPB, sphingoid base, SPBP, sphingoid base phosphate.

## Data Availability

The data presented in this study are available on request from the corresponding author. The data are not publicly available due to institutional rules regarding data sharing and privacy.

## Supplementary Materials

The following are available online at https://www.mdpi.com/2218-1989/11/2/93/s1, Table S1: Mean concentrations and standard deviations of sphingolipids measured, Table S2: Associations between protein intake and individual sphingolipid concentrations, Table S3: Associations between saturated fat intake and individual sphingolipid concentrations, Table S4: Associations between polyunsaturated fat intake and individual sphingolipid concentrations, Table S5: Associations between monounsaturated fat intake and individual sphingolipid concentrations.

## Abbreviations

BMI, body mass index; Cer, ceramide; CV, coefficient of variation; CVD, cardiovascular disease; FFQ, food frequency questionnaire; GalCer, galactosylceramide; GluCer, glucosylceramide; HDL, high-density lipoprotein; LDL, low-density lipoprotein; MET, metabolic equivalent task; MUFA, monounsaturated fatty acid; PUFA, polyunsaturated fatty acid; QC, quality control; SFA, saturated fatty acid; SM, sphingomyelin; SPB, sphingoid base; SPBP, sphingoid base phosphate; SPT, serine palmitoyltransferase; S1P, sphingosine 1−phosphate; T2D, type 2 diabetes.
